# New research tools suggest a “levels-less” image of the behaving organism and dissolution of the reduction vs. anti-reduction dispute

**DOI:** 10.3389/fpsyg.2022.990316

**Published:** 2022-08-29

**Authors:** John Bickle, André F. De Sousa, Alcino J. Silva

**Affiliations:** ^1^Department of Philosophy and Religion, Shackouls Honors College, Mississippi State University, Starkville, MS, United States; ^2^Department of Advanced Biomedical Education, University of Mississippi Medical Center, Jackson, MS, United States; ^3^Department of Neurobiology, University of California, Los Angeles, Los Angeles, CA, United States; ^4^Department of Psychiatry and Integrative Center for Learning and Memory, UCLA, Los Angeles, CA, United States; ^5^Department of Psychology and Integrative Center for Learning and Memory, UCLA, Los Angeles, CA, United States

**Keywords:** molecular and cellular cognition, ruthless reductionism, memory linking, C-C chemokine receptor type 5, head-mounted miniscopes, levels image, levels-less image

## Abstract

A kind of “ruthless reductionism” characterized the experimental practices of the first two decades of molecular and cellular cognition (MCC). More recently, new research tools have expanded experimental practices in this field, enabling researchers to image and manipulate individual molecular mechanisms in behaving organisms with an unprecedented temporal, sub-cellular, cellular, and even circuit-wide specificity. These tools dramatically expand the range and reach of experiments in MCC, and in doing so they may help us transcend the worn-out and counterproductive debates about “reductionism” and “emergence” that divide neuroscientists and philosophers alike. We describe examples of these new tools and illustrate their practical power by presenting an exemplary recent case of MCC research using them. From these tools and results, we provide an initial sketch of a new image of the behaving organism in its full causal-interactive complexity, with its molecules, cells, and circuits combined within the single system that it is. This new image stands in opposition to the traditional “levels” image of the behaving organism, and even the initial sketch we provide of it here offers hope for avoiding the dreary metaphysical debates about “emergence” and “downward causation,” and even the reduction vs. anti-reduction dispute, all dependent upon the familiar “levels” image.

## Ruthless reductionism guided the first 2 decades of molecular and cellular cognition

The neuroscience field of “molecular and cellular cognition” (MCC) began in the early 1990s. Gene targeting techniques, adapted into neuroscience from developmental biology, enabled experimenters to manipulate a single protein product that was part of some intra-or intercellular signaling pathway in cells in the brain, and to measure the effects of these manipulations on both cellular activities and organism behaviors. Silva et al. (1992a,b), in experiments, widely acknowledged to be the first ones published in this field, “knocked out” the gene for the *α* isoform of calmodulin kinase II at the embryonic stem cell stage of development in mice. They tracked the negative effects of this intervention on the induction of long-term potentiation (LTP) in mutant hippocampus tissue slices, and on learning in the intact mutants in the Morris water maze. Over the next decade, the spatial precision and temporal resolution of gene targeting in engineered mutants increased greatly; Silva et al. (2014) documents a number of these experiment tools and some key results in landmark MCC publications from its first 2 decades. Before the turn of the 21st century, Kandel and colleagues incorporated many of these early MCC findings into a “molecular model for the consolidation of the late phase of LTP and hippocampus-based long-term memory” (Abel et al., 1997, Figure 7, 623). Much of this original model is now current textbook neurobiology.

Reflecting metascientifically^1^ on these early MCC practices and results, and paying close attention to the language these scientists used in their experimental publications to describe their results, Bickle (2003, 2006) proposed that a novel type of reduction, “ruthless reduction,” was at work. Contrasted with popular accounts of reduction from the philosophy of science, including varieties of intertheoretic reduction, functional reduction, and then-newly described mechanistic reduction, Bickle argued that the ruthless reductionism implicit in MCC practices was a matter of intervening experimentally into increasingly lower levels of biological organization, and then tracking the effects of these interventions on behaviors *in vivo*, typically in rodents, using a variety of protocols widely accepted as operationalizing various cognitive functions. He argued that the most straightforward interpretation of what MCC scientists were doing in their experiments, and concluding in their discussion sections, was the direct reduction of cognitive functions to the intra-and inter-neuronal molecular pathways that the new gene targeting research tools were rendering experimentally manipulable. According to ruthless reductionism, the numerous other levels commonly thought to be interspersed between the molecular and the behavioral—especially the circuit and the “cognitive” (information-processing) levels—might be heuristically useful for finding new cellular and molecular mechanisms; but once these cellular/molecular mechanisms were found through rigorous MCC experimentation, the explanation of the behavior proceeded directly through those, embedded in the anatomical pathways that translated the activity of cells in the central nervous system out to muscle output and therefore measurable behavior.

Eventually Silva et al. (2014) provided a more extensive metascientific articulation of how molecular mechanisms account for behavior by defining the properties of “connection experiments” in MCC, which seek to establish causal relations between neurobiological kinds, including molecular mechanisms and single cognitive properties, for example. “Neurobiological kinds” had very broad scope in this context. Connection experiments in MCC could relate molecules, cellular physiology, circuit activities, or behavior. Within this more extensive account, “ruthless reduction” was modified into the typical MCC experimental practices to observe and manipulate hypothesized molecular mechanisms in “negative” and “positive manipulation” experiments that tracked the behavioral effects of molecular mechanisms in the well-known behavioral protocols for specific cognitive functions. Bickle and Kostko (2018) used this broader account to further modify ruthless reductionism, to include sets of experimental practices, well-illustrated by landmark MCC discoveries, by which multiple-experiment research programs are designed such that, if successful, the results of each component experiment will integrate directly with those of the other experiments. The goal of these multi-component research programs is to test activities throughout multiple-component causal pathways ultimately generating the behavior used to operationalize the cognitive function under investigation. Ruthless reductionism now incorporated the ongoing search for additional causal factors in the chains of causes revealed by multiple-experiment research programs, and the typical choice by MCC researchers to focus on components of the neurobiological kinds that connection experiments had already revealed to be parts of these causal chains. These later modifications of ruthless reductionism were also strictly metascientific hypotheses; they too were derived directly out of careful studies of landmark MCC publications, including the experiment designs, reported findings, and conclusions offered in discussion sections of landmark MCC experimental publications.

No one should deny that the reductionist approach at work over the first 2 decades of MCC has been extremely successful, and extremely valuable toward understanding how different aspects of brain biology contribute to a given cognitive process or behavior. The MCC track record from the early 1990s through the 2010s is a testament to those successes. However, it also seems apparent that even this sophisticated reductionist approach, when supplemented with increasingly sophisticated experiment tools built on the latest technological marvels, can miss important components and processes at work in systems as complex as the mammalian, much less the human brain. Nothing mystical or magical motivates this worry. Reductionist approaches of necessity divide complex phenomena, cognitive or otherwise, into their basic components, be those components molecules, cells, circuits, or behavior. They then carefully manipulate these components *individually,* in a tightly controlled fashion, to investigate that component’s causal contribution to the system’s behavior. This “decomposition” approach is necessary for exploring whether the factor in question is or is not a part of the causal nexus generating the system’s target behaviors. But approaching a system’s causal structure in this reductionist fashion inevitably leaves open the possibility that some key components in the complex chains of interacting, interconnected causes might still remain unnoticed or unspecified. This worry is exacerbated by the typically incomplete temporal, cellular, and circuit precision of early MCC tools, which made it difficult to integrate molecular, cellular, and circuit mechanisms in explanations of behavior.

There is also a deeper philosophical worry about reductionism, to which the ruthless reductionism implicit in early MCC experimental practices is not immune. This worry has been stressed in numerous recent publications by Michael Silberstein, Philippe Huneman, and Sara-Lee Green and Robert Betterman, and others. It holds that there are system-level properties that can only be understood by investigating the system “holistically,” and which require their own type of nonreductive, emergentist explanations. These authors stress such properties investigated by different sciences, but neural system properties are common to many of their arguments.

If only MCC experimenters could manipulate a specific molecular component of the brain, e.g., some specific gene or molecule, in a specific collection of cells (neurons, glia, etc.) and then observe not only how that manipulation affects the system’s overall behavior, but also somehow simultaneously how that manipulation affects other components throughout the system, e.g., activities in cellular networks or brain circuits. And if only these manipulations had the temporal and cellular resolution to permit for the first time, the investigation of the genetic or molecular component at time scales that are compatible with cellular and circuit operations that are relevant for behavioral output. Such an experimental tool might address the worries about even sophisticated reductionist approaches we just sketched. Results from using this new tool might even suggest an alternative to the traditional categorization of behaving systems in terms of “levels of analysis”; it might offer us a novel “levels-less” image of the inevitable causal interdependence between an organism’s behavior, circuit activities, and the cellular and molecular components that make up its brain.

As we will report here, new research tools have become available to do exactly the kinds of manipulation and observation experiments we just described. The increasing use of these tools has led one prominent MCC laboratory to assert the emergence of a new field, “molecular systems neuroscience” (Shen et al., 2022a). These new imaging and manipulation tools and the results they are generating carry considerable implications for the reduction vs. anti-reduction dispute. All discussions of reduction vs. anti-reduction in science occur against a backdrop of some account of distinct “levels”: of analysis, of description, of experimental investigation, of mechanisms, or of reality. It is whether the reduction relation holds between components of these distinct “higher” and “lower levels” that reductionists and their opponents dispute. What is especially philosophically intriguing about these new studies in MCC is that they suggest an alternative image to the “levels” picture of the behaving, interacting system; and this alternative, “levels-less” image opens the possibility of sidestepping the reduction versus anti-reduction dispute. To a first approximation, what we find in these recent MCC studies is an image of the organism in its full causal-interactive complexity, with its molecules, cells, and circuits combined into the single system that it is. Our goal in the final section below is to provide a first sketch of this new image, and even to diagram it opposite to the traditional “levels” diagram. Even our initial sketch of this new image suggests that we can transcend the reduction vs. anti-reduction dispute, along with the dreary metaphysical arguments about how entities and processes across “levels” causally interact or relate. The “levels” metaphor ultimately traces back to medieval disputes about “levels of Being,” rooted in neo-Platonism and theology. Surely, we can all welcome a 21st century scientific worldview freeing itself from that arcane, ancient scaffolding.

## A case study from recent MCC that uses some of these emerging technologies

To present these new experiment tools in some detail, we next describe a recent example of research using them. Shen et al. (2022b) investigated how the mammalian brain links two individual memories acquired close in time to generate a novel mnemonic structure, “linked memories,” that support adaptive behaviors. “Mnemonic structures” are theoretical constructs that the brain creates and uses to relate information linked by different dimensions of experience, including time, space, and perceptual or conceptual similarities (de Sousa et al., 2021). Recent studies in rodents had demonstrated that the linking of memories acquired close in time (e.g., 5 h apart) depends on the percentage of overlapping neurons encoding each memory, with linked memories sharing more encoding neurons than non-linked ones (Cai et al., 2016; Rashid et al., 2016).

One possibility for why memories become linked when acquired in close temporal proximity is the “allocate-to-link” hypothesis, which is based on the observation that after a learning event, neurons involved in memory encoding have, for a period of time, increased activity of the cAMP response element-binding protein (CREB), a gene transcription modulator, and consequently a temporary increase in intrinsic excitability (Silva et al., 2009). Since more excitable neurons are more likely to be allocated for memory encoding (Han et al., 2007; Rogerson et al., 2014; Yiu et al., 2014; Josselyn and Frankland, 2018), subsequent and related events that occur close in time to the first event will have a higher likelihood of engaging neurons that were involved in encoding the first event. This way, two independent memories can become linked *via* their overlapping and shared neuronal ensembles. Future retrieval of one memory will increase the likelihood of retrieving the other due to the reactivation of the neuronal ensembles of both memories. However, while CREB expression and neuronal excitability have been thought to open the window for memory linking, it has not been known whether this window is closed by a passive process, or whether there is an active mechanism that closes the temporal window for memory linking. Shen et al. (2022b) addressed this question by investigating the critical role of C-C chemokine receptor type 5 (CCR5) as a negative regulator of CREB activity and neuronal excitability (Shepherd et al., 2013; Zhou et al., 2016). CCR5 is a major chemokine receptor that had been extensively studied in the context of HIV infection (Brelot and Chakrabarti, 2018). More recently, Zhou et al. (2016) demonstrated the role of this receptor in suppressing CREB signaling and affecting neuronal plasticity following learning, and Joy et al. (2019) then showed that the levels of this receptor can dynamically change in the brain. Given the role of CCR5 as a suppressor of CREB activity and neuronal excitability, and in turn, their involvement in memory linking, the results presented above raised the tantalizing possibility that changes in CCR5 levels or activity following learning may affect memory linking.

Shen et al. (2022b) first replicated the behavioral finding that mice link the memories of two different spatial contexts when the contexts are explored on the same day (e.g., 5 h apart), but not if they are explored on different days (e.g., 2 days apart; Cai et al., 2016). Mice were allowed to explore one novel context (context A) for 10 min, and then, 5 h or 1–7 days later, they explored a second novel context (context B) for 10 min. Two days following the second exploration, mice received a mild foot shock immediately after entering context B and their conditioned response, freezing level, was subsequently measured upon re-exposure to context A, context B, and a novel context. Freezing is an innate response that rodents display when presented with a threatening stimulus that may elicit fear. The animal crouches and remains motionless except for breathing. In this behavioral paradigm, freezing indicates that the mouse formed an association between a particular context and the aversive foot shock. This paradigm is well established in the MCC field and mice usually develop a conditioned response (freezing) specifically to the context where they received the foot shook and not to other contexts (i.e., mice can discriminate between an aversive context and a neutral one). Interestingly, in the linking experiments described above, mice that had visited both contexts 5 h apart froze for the same amount of time when re-exposed to both contexts A and B, although they had never been shocked in context A. In contrast, mice that visited the two contexts 7 days apart only froze when re-exposed to the context where they received the shock. These results indicate that although mice in the 5-h group had never received a foot shock in context A, they were displaying the same conditioned response observed in context B, as if retrieval of the memory for context A induces the retrieval of the linked memory for context B, where mice were indeed shocked. Importantly, none of the groups displayed significant freezing when exposed to the novel context in the test phase, excluding the possibility of simple fear-to-context generalization.

Shen et al. (2022b) also characterized the expression of CCR5 messenger RNA (mRNA) in the mouse dorsal hippocampus, a brain region involved in memory linking (Cai et al., 2016). They observed that under baseline conditions, most CCR5 mRNA expression is found in microglia cells, with only some limited expression in neurons. However, 6–12 h following a learning event, expression of CCR5 mRNA dramatically increases in dorsal hippocampus neurons, especially in neurons involved in memory encoding, the so called “engram cells,” (Josselyn and Tonegawa, 2020). This increase in the expression of CCR5 mRNA was accompanied by a similar increase in the expression of CCL5 mRNA, one of the ligands of this receptor, suggesting a potential activation of the receptor during this time frame. However, these increases in mRNA levels do not necessarily translate into activity of the CCR5 receptor and classical MCC tools do not allow researchers to track the activity of CCR5 with the temporal and cellular resolution necessary for testing its involvement in memory linking. To address this problem, the authors created a new tool, CCR5-iTango2, to probe the activity of CCR5 receptor *in vivo* during a learning event. This system is based on the iTango tool, a sophisticated molecular system first reported in *Nature Methods* in 2017 (Lee et al., 2017). iTango is a ligand-and light-gated labeling system whereby neurons express a fluorescent reporter protein if the target molecular activity occurs within them while these cells are exposed to blue light. This optogenetic “light switch” insures that the activation observed actually took place within a precise time window marked by blue light activation. This enables the identification of the molecule’s activity in specific populations of cells during specific timepoints using immunohistochemistry techniques. Using this approach, Shen et al. (2022b) demonstrated that CCR5 is indeed highly activated for 6–24 h after the learning event, particularly in dorsal hippocampus neurons involved in memory encoding (“engram neurons”). This experiment is a good illustration of how novel tools in MCC are allowing researchers to probe the activity of molecules with unprecedented cellular and temporal resolution that is essential to understand their activity in the integrated context of specific cell, circuit, and behavioral activity.

Given the role of the CCR5 receptor in suppressing CREB signaling, and the role of CREB in memory allocation, the authors hypothesized that activation of CCR5 during this period of time could be involved in closing the window for memory linking. To test this possibility, the authors exposed mice to context A and 4 h later infused CCL5 into dorsal hippocampus to activate CCR5 receptors. One hour after infusions, mice were exposed to context B and underwent the memory linking behavioral paradigm as described above. Remarkably, overactivation of CCR5 by CCL5 infusions prevented memory linking without disrupting fear memory for context B, suggesting that this signaling pathway is able to selectively modulate memory linking. Although still informative, this classical MCC approach suffers from low temporal and cellular resolution since ligand infusions can affect molecules for long periods of time and lack cellular or circuit specificity since the ligand can diffuse in the brain and affect multiple circuits in adjacent brain areas. To gain better cellular, circuit, and temporal resolution, the authors built a novel optogenetic tool, Opto-CCR5. With this tool, CCR5 can be activated by simply using blue light. Neuroanatomical analyses can also precisely confirm where CCR5 was activated. A key component of Opto-CCR5 is a receptor protein from the “Opto-XR” family, a group of opsin-receptor chimeric proteins developed through the fusion of a light sensitive receptor protein (rhodopsin) and different G protein-coupled receptors (GPCRs; in this case CCR5; Airan et al., 2009). Using the same memory linking behavioral paradigm, the authors showed that activation of Opto-CCR5 before exposure to context B, 5 h after exposure to context A, led to an impairment of memory linking. Thus, with two very different tools, the authors were able to demonstrate that CCR5 activation is *sufficient* to close the temporal window for memory linking. The new tools are giving much more than additional precision and specificity: they are allowing the design of experiments that explore and test the interactions between molecules, cells, circuits, and behavior in ways that were unthinkable even 10 years ago. This precision and specificity have freed MCC researchers from the previous reductionist logic that implicitly or explicitly dominated the field.

The authors then tested whether CCR5 activity is *necessary* for closing the memory linking window. To this end, the authors used mutant mice engineered to lack the CCR5 or CCL5 genes and they also expressed a short hairpin RNA (sh-RNA) to decrease CCR5 expression in dorsal hippocampal CA1 neurons of wild type mice. shRNA is a bioengineered artificial RNA molecule designed to inhibit the expression of a desired gene. In all three experiments, mice were exposed to context B (and the foot shock) 2 or 7 days after initial exposure to context A, a time frame over which mice do not show memory linking. Remarkably, all three manipulations not only decreased CCR5 or CCL5 expression, but they also dramatically expanded the temporal window for memory linking since the mice with these manipulations froze just as much in the never-shocked context A as they froze in shocked context B that they saw either 2 or 7 days apart, times when normally mice fail to link memories. These results indicated a critical causal role for the CCR5/CCL5 system in memory linking by demonstrating that increasing or decreasing CCR5 activity directly impairs or extends (respectively) memory linking.

To understand how CCR5 could be affecting cellular and circuit properties relevant for memory linking, Shen et al. (2022b) used miniature head-mounted fluorescent microscopes (miniscopes; Ghosh et al., 2011; Cai et al., 2016), to image the activity of many individual neurons (>300 per animal) in the dorsal CA1 region of the hippocampus, in real time while mice were engaged in the memory linking behavioral paradigm. Specifically, Shen et al. (2022b) monitored the level of intracellular calcium ions (a proxy for neuronal activity) with a genetically-encoded fluorescent molecular reporter (GCaMP6f,) engineered to detect cytoplasmic free calcium ions in activated neurons. With this novel technology, the authors were literally observing neuronal activity (or lack thereof) throughout the dorsal CA1 region of the hippocampus in behaving mice.

Consistent with the hypothesis that the overlap between memory ensembles in the dorsal CA1 regions of the hippocampus determines memory linking (Cai et al., 2016), the authors showed that a manipulation that expanded the temporal window for memory linking (e.g., CCR5 knockout) also expanded the temporal window in which they saw higher overlap between the CA1 memory ensembles for each of the two contexts in the memory linking experiment. The use of miniscopes in these memory linking experiments was crucial since it allowed the authors to determine the active neurons that were present in both memory ensembles (the overlap neurons) with a precision and with time windows (e.g., 7 days) that were simply impossible with previously used MCC technologies, such as with intracranial recording electrodes. The authors further observed that when wild-type mice explore the two contexts 5 h apart, the overlapping activated cells had significantly less CCR5 expression than do non-overlapping cells, indicating that this receptor might be directly modulating the extent of ensemble overlap. To directly test this last hypothesis, the authors used the Opto-CCR5 system to selectively activate the CCR5 pathway with blue light in specific neurons before the mice explored a new context. Using immunohistochemistry techniques, they demonstrated that those neurons with optogenetic activation of CCR5 (neurons with Opto-CCR5) were excluded from memory encoding, a result consistent with the idea that delayed expression of CCR5 in neurons engaged by the first memory excluded these neurons from also participating in the encoding of the second memory, thus closing the temporal windows for memory ensemble overlap and memory linking.

Finally, the authors used neuronal recordings in brain slices to show that increases in CCR5 activity with CCL5 resulted in lower neuronal excitability, a finding that explains why higher activity levels of this receptor cause decreases in memory allocation, and consequently lower ensemble overlap and loss of memory linking. Together, this impressive set of convergent and consistent findings involving molecular (CCL5/CCR5) cellular (neuronal excitability), circuit (memory allocation; memory ensemble overlap in CA1), and behavioral phenomena (the memory linking paradigm) provide a compelling example of how studies involving multiple entities typically defined at different levels of analyses not only provide a more complete explanation of behavioral phenomena such as memory linking, but they also help to strength the convergent and consistent findings that are at the basis of developing explanations of brain phenomena. Manipulations of CCR5 affected excitability, memory allocation, memory ensemble overlap, and memory linking in a consistent manner. For example, manipulations that increased CCR5 activity decreased neuron excitability, decreased memory allocation, reduced memory ensemble overlap (measured through the miniscopes), and prevented memory linking (measured behaviorally). The CCR5 manipulations not only tested the connections between this receptor and each of these other four phenomena, but they also tested predictions of the allocate-to-link hypothesis that these four phenomena are causally connected (Silva et al., 2009).

In the last section of the paper, Shen et al. (2022b) showed that increases in CCR5 also accounted for the loss of memory linking in middle-aged mice (Cai et al., 2016). Previous results had shown that aging can alter chemokine signaling in the brain (Felzien et al., 2001). The authors first measured the levels of CCR5 and CCL5 mRNA in middle-aged mice at baseline conditions and observed a significant increase in the expression of both genes compared to young mice. Moreover, 3 h following learning, middle-aged mice showed a sharp increase in CCL5 expression, which was earlier than the peak observed in young adult mice (6–12 h). This observation raised the possibility that an early increase in CCR5 signaling could be responsible for the impairment in memory linking in middle-aged mice. Remarkably, middle-aged mice with a CCR5 knockout were able to link memories encoded 5 h apart, indicating that CCR5 signaling could indeed be responsible for the age-related deficits in memory linking. To further test this hypothesis, Shen and collaborators infused Maraviroc, an FDA approved CCR5 antagonist used in the treatment of HIV, into the hippocampus of middle-aged mice and showed that this treatment was sufficient to reverse the loss of memory linking in these mice. These results may have significant clinical implications since Maraviroc is an FDA approved drug and could be used in clinical trials to determine whether it is effective in treating deficits in memory linking associated with aging and psychiatric disorders.

The development of new technologies like miniscopes, iTango2, and Opto-CCR5 is allowing MCC and other neuroscience researchers to transcend constraints imposed by traditional reductionist experimental approaches that in part were imposed by technical limitations of previous approaches. With the increased temporal, cellular and even sub-cellular precision of the new measurement and manipulation techniques, it is now possible to not only test more precisely ideas about the role of molecules in behavior, but also to meaningfully test the impact of specific circuit changes caused by those molecular manipulations in behavior in ways that were unthinkable just a few years ago. Thus, these powerful new tools are helping neuroscientists to study how different biological components in the brain (e.g., a specific gene, group of cells, or targeted circuit) interact to generate brain states and behavior.

Here we have summarized the experiments in just a single recent publication from one lab, but they illustrate a number of these new imaging and manipulation research tools that are transforming research in neuroscience. The crucial next step is to both expand and shift the scope of studies, from what are traditionally assumed to be a single level of analyses to studies that are instead focused on how different phenomena classified traditionally at different levels of analyses interact to generate brain states and behavior. This collection of new tools makes such investigations possible. For example, using light-sheet microscopy and immediate early gene expression, researchers can now routinely image the entire brain of a mouse at cellular and even sub-cellular resolution, and thus map neurons across the brain that are active during specific behaviors (DeNardo et al., 2019). Likewise, identification and genetic profiling of neurons with specific roles in behavior (e.g., neurons forming overlapping ensembles in memory linking) open the door for a new type of understanding of the heterogeneity of neuronal populations working together across the brain to generate and modulate behavior. Finally, a large number of recent studies have consistently demonstrated that brain states and behavior not only depend on neurons, but also on a number of other cell types in the brain, including astrocytes, microglia, and oligodendrocytes. Ultimately, approaches that combine brain-wide imaging with single cell (even sub-cellular) resolution, with temporal and site-specific manipulations of molecules, different cell types, and circuits in the behaving animal will be key to understanding how all of these components interact causally to give rise to brain states and behavior.

## A “levels-less” image of the behaving organism and a way around all of the philosophical conundrums that the levels image generates?

The new research tools we illustrated in the previous section promise scientific progress. But might the results that stem from their use also revamp our traditional image of the behaving organism and the organization of its interacting components; and thereby lead to philosophical progress as well?

Since the mid-20th century, the reduction vs. anti-reduction dispute has occurred explicitly against a backdrop of a “levels” account of reality, or of scientific inquiry, or of both. For Oppenheim and Putnam (1958, 9), the distinct levels were the universes of discourse of the various branches of science: social groups, multi-cellular living things, cells, molecules, atoms, and elementary particles. According to Oppenheim and Putnam, the key relationship holding between elements across these levels was micro-reduction of the higher-level elements to those at the next level down. For Ernest Nagel, the levels at work in “heterogeneous” cases of intertheoretic reduction, where the “primary theory” reduces a “secondary theory” even though the latter contains descriptive terms not present in the former, are those of macroscopic phenomena dealt with by the secondary theory and “a microscopic constitution for those macroscopic processes” postulated by the primary theory (1961, 340). “Intertheoretic reduction” for Nagel was deduction, of the logical structure of the secondary (reduced) theory from that of the primary (reducing) theory, supplemented with whatever “conditions of connectability”^2^ were necessary to link descriptive terms of the secondary theory not present in the primary theory to terms of the primary theory, plus whatever limiting assumptions or boundary conditions were necessary on the primary theory owing to its typically broader explanatory scope. In relatively smooth intertheoretic reductions, the macroscopic entity denoted by some term from the secondary theory (e.g., heat) linked in some cross-theory condition of connectability were deemed identical to the microscopic entities denoted by the related terms from the primary theory (e.g., mean kinetic energy of the system’s constituent molecules). Reductionists have always claimed to hold a *prima facie* metaphysical advantage over anti-reductionists, insisting that reduction implies (or at least provides evidence for) cross-level identities. Most anti-reductionists have sought to provide some cross-level relationship logically weaker than reduction, yet not committed to some kind of spooky dualist status for the non-reducible entities or processes.

**Figure 1:**
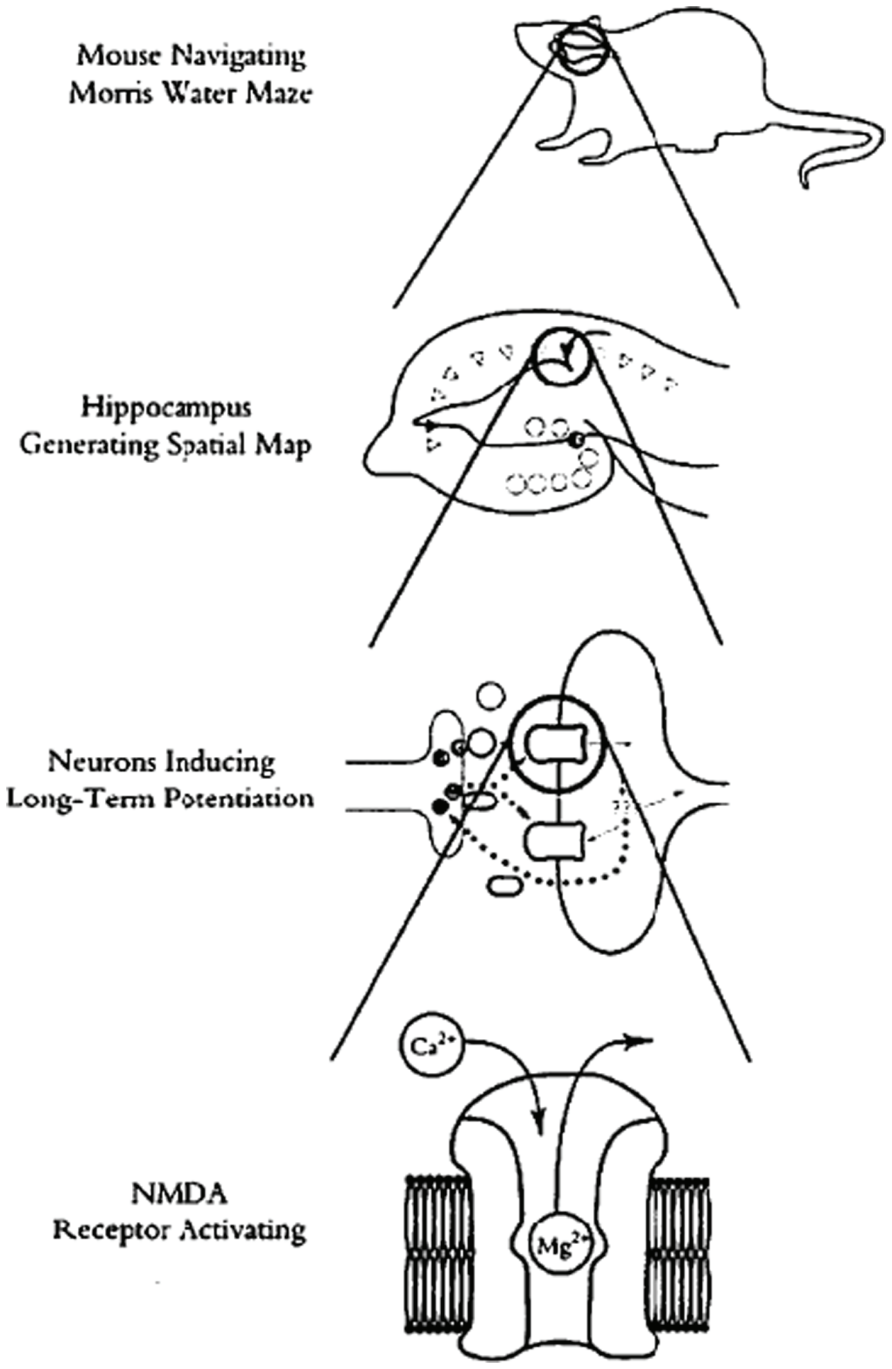
The classic levels image of the behaving organism (rat navigating the water maze). (Original caption: “Levels of spatial memory”) Reprinted with permission from Craver (2007), Figure 5.1, 166.

Most recently, “new mechanists” have introduced an account of “levels” into these discussions that they claim to be less contentious than previous ones. In his comprehensive account of neuroscience from the “new mechanist” perspective, Craver (2007) spends an entire chapter (chapter 5) providing a “field guide” to levels in the philosophy of science. Ultimately, he elaborates and defends a novel “levels of mechanisms” account. The “next level down” from any given target system are that system’s components, the individual dynamics of those components, and their organization that generates the system’s input–output behavior. The entire system is thus a nested hierarchy of such mechanisms-within-mechanisms, as the mechanisms at the next level down that compose the higher-level system are themselves composed of components, their dynamics, and their organization at the next level down. Figure 1 is much discussed diagram of Craver (2007, Figure 5.1, 166) of the nested hierarchy of mechanisms-within-mechanisms of a rat navigating a water maze. Interestingly, mechanists who share this basic account of levels have differed about whether it is reductionist or not. Bechtel (2009) advocates it explicitly as an account of “mechanistic reductionism”; Craver (2007) remains ambivalent.

**Figure 2:**
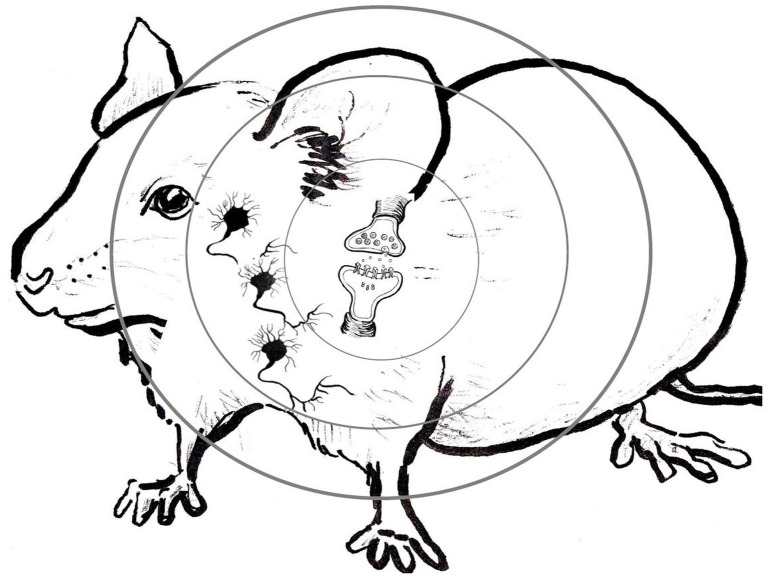
The “outwards” levels-less image of the behaving organism suggested by results using the new research tools of molecular and cellular cognition (MCC), with myriad causal relations obtaining between intra- and intercellular molecular pathways (innermost concentric circle), cells and networks of cells (middle concentric circle), and the behaving organism (outermost concentric circle; Original artwork by Caroline Cooper.).

Is philosophy of science inevitably stuck with some vexing “levels” concept, in one form or another, and so with the inevitable and seemingly unresolvable disputes between those who insist that higher levels reduce to lower levels, and those who deny reduction and insist on some weaker cross-levels relationship? The recent directions in MCC research we illustrated in our case study in the previous section, guided by the new research tools, suggests a new image of the behaving organism, a “levels-less” one in which distinctions between “levels” need not sidetrack us into solutionless metaphysical disputes. Instead of picturing the behaving organism from its molecular level “up” to its cellular level, then “up” to its circuitry or network level, and finally “up” to the behaving organism level itself, as illustrated in Figure 1, and then wondering how components or ongoing activities at any one of these levels relate to those at others, picture instead scrunching the entire image *down*, “level” *within* “level,” into the single interacting system that it is, replete with molecular pathways *in* cells, cells *in* networks, and networks *in* the behaving organism itself. Then start with some activated intracellular molecular pathway in some central neurons, the usual target of positive and negative manipulations in MCC experiments. Then, move seamlessly from that intraneuronal molecular signaling pathway *outward*, first to the individual neurons whose bilipid membranes encase those manipulated molecular components and pathways. Continue to move seamlessly *outward* from activity in those individual neurons, into the wider cellular circuits they are part of; not only with other neurons, but also with glial, endocrine, immune, and muscle cells—with cells of all other types of tissues with which those neurons form active interacting circuits. Finally, move further seamlessly *outward* to the behaving organism itself, inside the skin of which all of those cellular circuits are active, of that single, marvelously interactive system. Some of the active molecular pathways encased within cellular membranes move molecules across these membranes selectively, into other cells. Those cells combine into circuits that actively communicate with one another, typically *via* molecular exchanges and interactions. Neural circuitries connect with sensory receptors of various sorts, which transform environmental energy of specific kinds into cellular activities, while motor neurons in these circuits communicate directly with muscle tissues to contract muscle fibers against the calcium frames (the bones) to which tendons connect these muscles. All of these components—the interacting molecules, the cells, the circuits, the sensory receptors, the muscles, and bones—are interacting elements of one and only one system, the behaving organism. Figure 2 diagrams this alternate image to the classic “levels” image of Figure 1.

The exquisite temporal and spatial specificities of the new technologies guiding recent MCC research allow researchers to transcend the ruthless reductionism of previous MCC work, since these technologies allow for meaningful integrated studies, simultaneously and in real time, across what tradition clumsily separates as various “levels.” For example, early MCC studies with alpha CaMKII were very specific at the molecular level. They deleted the alpha CaMKII gene without deleting others. But the widespread effects of this single deletion in multiple circuits at different developmental stages made cellular and circuit studies very difficult if not impossible to run. By contrast, the precision and specificity of the new tools used in the CCR5 studies described in section 2 (OptoCCR5, head-mounted miniscopes) complement the use of more traditional MCC tools such as the CCR5 knockout mutant mice. They make analyses and interpretation of circuit properties, such as CA1 neuronal ensemble overlap, measurable in real time, compelling, and meaningful. The use of miniscopes permitted meaningful and long-term imaging of the activation patterns of entire neuronal ensembles in freely behaving animals. “Long-term” is an important addendum here, because before miniscopes were developed, MCC researchers could only observe neuronal activity changes in freely behaving animals (e.g., mice) after specific molecular manipulations by using tools such as intracranial electrodes. Miniscopes permit experimenters to record from more neurons in key circuits and for longer periods of time. These advances are crucially important. With electrodes, one could never even be sure that the same neurons were being studied as time went on. The molecules of intracellular signaling pathways in specific neurons, the effects on circuit activities to single neuron resolution, and the behaving animal can now be manipulated and monitored simultaneously, in individual experiments. Hence our first attempt to sketch the levels-less image of the behaving organism that these new tools are revealing.

Thinking upwardly in terms of different “levels,” even in terms of seemingly innocuous “levels of mechanisms,” only clouds our emerging capacities to manipulate and track interacting components of the entire system, simultaneously and in real time. Thinking outwardly instead, from the molecular pathways in specific cells, to the cell assemblies those cells are part of, to the brain networks those cell assemblies connect up, and finally to the behaving system itself, which these new precision molecular intervention and circuit-activity imaging technologies now permit, all at once, seems to absolve us of any need to separate the organism into distinct “levels.” The power of this intriguing alternative image of the behaving organism is that all of the philosophical conundrums that the levels-image generates no longer demand answers. Start with the long-standing reduction vs. anti-reduction dispute. Armed with this new image of the behaving organism and its myriad interacting components, we now face no mysteries about how intervening into a specific molecular component in selected neurons can directly affect specific network or circuitry activities in a specific brain region, or even the entire organism’s behavior. Because with the new MCC experiment tools, we can now *observe* these network or circuitry effects directly, and at the same time, we are observing the effects of our molecular manipulations on the organism’s behavior. No multitude of “levels’ is being “leaped in a single bound,” from molecules to behaving organism. There is just the single system that is the behaving organism and its myriad interacting components. No elaborate cross-levels metaphysics is needed to “bridge multiple levels,” since the new image suggested by results garnered using these new research tools does not relegate these components into distinct levels.

In the section “Ruthless reductionism guided the first 2 decades of molecular and cellular cognition,” we mentioned two worries that the requisite reductionist focus on single components of the behaving system generates. One was that this focus inevitably leaves out too many possible causes contributing to a complex system’s behavior that have yet to be investigated, especially those of broader systems that the single manipulated component is a part of. The new MCC experiment tools we illustrated in the section “A case study from recent MCC that uses some of these emerging technologies” are tailor-made to investigate contributions of exactly these kinds of circuitry components, simultaneous with the molecular interventions and the standard behavioral measures. The second worry was the challenge that systems level properties can only be explored by investigating the system “holistically,” and requires a special kind of explanation. While it is true that the new network-level imaging technologies like head-mounted miniscopes are a novel addition to MCC research, their use in MCC experiments is just one part of complex experimental designs that combine these measures in real time with precise molecular manipulations and behavioral measures of cognitive functions. All of these molecular, cellular, network, and behavioral techniques merge together in studies like the one we described in the section “A case study from recent MCC that uses some of these emerging technologies,” in a fashion that no reductionists or anti-reductionists ever previously considered. And now no special explanations are required for the network or system properties, when they are investigated in the fashion illustrated by our example.

As philosophical discussions can be, our initial attempt here to sketch an alternative to the standard levels-account of the behaving organism might seem annoyingly abstract; some might worry that we have construed this new image mostly negatively so far, as not the familiar levels image.^3^ So let us try to flesh it out further and state its philosophical advantages in more concrete, positive terms. Consider a hypothetical experiment. Suppose we engineer CCR5 knock-out mice, which would normally increase their memory linking capacities. But suppose we couple these mutant mice with some artificial method for decreasing activity in specific neurons in the dorsal hippocampus to prevent neuronal ensemble overlap. Presumably, we would thereby inhibit memory linking in the manipulated mutants.^4^ Within any levels-framework, even within the innocuous nested-hierarchy-of-mechanisms-within-mechanisms framework (Figure 1), we would seem here to have generated a cellular-level mechanism, namely, our artificial method for decreasing cellular activity and ensemble overlap. That mechanism seems to override our molecular pathways-level mechanism for increasing memory linking, *via* our CCR5 gene knock-out mutation. On the traditional “levels” picture, this combination of experimental manipulations would seem to be an instance of a higher-level mechanism causally overriding a lower-level one. And we thereby generate the logical and metaphysical conundrums tied up with “downward causation,” with the higher-level components and activities in their capacities as (or *qua*) higher level components, causally affecting the lower-level processes.^5^ Or we face articulating the problematic cross-level identities that reductionists champion, but which they rarely articulate in any detail (Exactly which activities in which intercellular molecular pathways are our artificial cell-level interventions identical to?). But when we replace the mechanists’ nested hierarchy of mechanisms-within-mechanisms image with the single interacting causal system we are sketching here (Figure 2), these logical and metaphysical mysteries vanish. There are no levels that need to be crossed. The dorsal hippocampus neurons whose activity we envision causally decreasing experimentally contain those CCR5 molecular pathways, so do the circuits of interacting neurons and the behaving organism itself. All components of the system are in ongoing causal flux. No special “downward” causes from higher to lower “levels” of the system’s components are needed; just ordinary causation of the sorts that connection experiments in science routinely provide evidence for.

Finally, what about the anti-reductionist worry that mapping neuron activity across the brain at single-cell resolution provides a uselessly complex data set that cannot be understood without appealing to organizational principles, and with these principles necessarily cashed in terms of distinct levels?^6^ Investigating biological systems using experimental tools that measure or produce narrow changes in specific biological kinds at any given time is a necessary evil in our endeavors to understand the behaving organism in all of its causal complexity. There simply is no absolute way, currently or for the foreseeable future, to capture completely all changes occurring at once inside an organism as it behaves. However, a new capacity to map brain-wide changes of experimentally-induced neuronal activities, against a background image that assumes no intrinsic levels of analysis and no hierarchal streams of causation, has the benefit of not compartmentalizing the changes we can affect, and now observe and measure, as separate from all the other biological kinds that make up the behaving organism. In this sense, the mapping of active neurons within brain circuits while manipulating a step in a molecular cascade in some of those neurons is an excellent example to support our sketch of a level-less image of the behaving organism.

As an example, to map neuron activity across the brain at single-cell resolution, it is now routine to observe the expression of specific immediate early genes as a proxy for individual neuronal activity, because we now know that these genes are only expressed once neurons fire above a certain rate threshold; we know that firing rate induces the changes in those genes’ expression. Using the levels image, this would suggest that a higher-level kind—a neuronal or circuit feature—directly affected changes at a lower level—the mechanisms of immediate early gene expression; thus implying a direction of causation from higher to lower levels. However, one can readily see that in order for a neuron to fire in the first place, it needs activities in the genetic and molecular components that constitute it. Upon a synaptic input, synaptic receptors are activated by neurotransmitters at the postsynaptic terminal, ions flow through ion channels in the cell membrane leading to changes in membrane potential and the neuron may fire an action potential that propagates down its axon. What caused the neuron to fire? The synaptic input? The neurotransmitter receptors? The ion channels? Each of these “lower level” components is reasonably considered to be part of the causal chain that makes the neuron fire. In turn, neuronal firing will change immediate early gene expression, which can change synaptic responses *via* changes in synaptic plasticity and membrane excitability, which in turn might change the way the neuron fires the next time. So, on a levels view, there seems to be a loop of causality between higher and lower levels. These loops pose challenges to reductionist views, but they also saddle anti-reductionists with explaining all of these multi-level causal interactions in a scientifically legitimate way.

Instead, if we understand the entire organism as a single system in which all components interact in a single plane of causal interactions, we can start to appreciate the constant flux of interdependencies that make up all the material components of the brain. Ions, molecules, cells, circuits, and the entire brain are parts of a single construct that we call a living organism. To make reductionist claims about any of these components is to ignore all the magnificent complexity that exists between all these biological kinds. To make anti-reductionist claims invites imputing activities that cannot be cashed out scientifically. The new MCC experiment tools presented here, although far from perfect, allow us to take the first steps toward a new levels-less image of the behaving organism. An ideal future scenario would be able to observe and manipulate each of the different components at timescales relevant for each process, and to understand their joint impact on all the other components. Obviously, we are not there yet. But the new tools MCC researchers now have and the results they are starting to generate suggest the levels-less image of the behaving organism that we provide a first sketch of here. It obviates the need for philosophical accounts of how biological kinds at different levels interact. The behaving animal is a single, complex system of muscles contracting against skeletal frames, receptors being activated, neurons firing, genes being activated or repressed, molecules interacting, even atoms moving from one place to another, with all of these in ongoing causal interactions. But there is nothing mysterious about these components or interactions calling out for special philosophical theorizing. They are just nature’s building blocks, interacting in ways optimized by natural selection to promote the evolutionary fitness of the organism. And our levels-less picture better reflects our increasing capacities to both intervene and image activities simultaneously in many of these components than does the traditional levels image.

The levels metaphor entered into Western intellectual discourse from speculations about “levels of being” in medieval theology. Results obtained using the new research tools that are revolutionizing recent MCC research, such as the ones we described in the section “A case study from recent MCC that uses some of these emerging technologies,” suggest a new image of the behaving organism and its myriad interacting components that may finally let us lay that antiquated “levels” notion thankfully to rest. With the levels notion also go the many philosophical puzzles it has generated. The reductionism vs. anti-reductionism dispute is one of those puzzles. Does more need to be said to further flesh out this alternate image, and about how results using these new MCC experiment tools contribute to that fleshing out? Absolutely! Ours is only a first attempt to draw out this alternative image from ongoing science. But its promises seem well worth the effort. A useful next step could be an account of how this new image might generate different kinds of experiments that the traditional levels-image obscures. Our hypothesized experiment just above could be a first step toward providing that.

## Data availability statement

The original contributions presented in the study are included in the article/Supplementary material, further inquiries can be directed to the corresponding author.

## Author contributions

ASi and ASo contributed to the research and publication of the scientific results described in the section “A case study from recent MCC that uses some of these emerging technologies.” JB, ASo, and ASi contributed equally to the writing of this manuscript. All authors contributed to the article and approved the submitted version.

## Funding

This work was supported by grants from the NIMH (R01 MH113071), NIA (R01 AG013622), NINDS (RO1 NS106969), and from the Miriam and Sheldon G. Adelson Medical Research Foundation to ASi.

## Conflict of interest

The authors declare that the research was conducted in the absence of any commercial or financial relationships that could be construed as a potential conflict of interest.

## Publisher’s note

All claims expressed in this article are solely those of the authors and do not necessarily represent those of their affiliated organizations, or those of the publisher, the editors and the reviewers. Any product that may be evaluated in this article, or claim that may be made by its manufacturer, is not guaranteed or endorsed by the publisher.

## Footnotes

^1^Bickle (2003) introduced this term and has used it since to denote a method for studying particular concepts or notions in science by carefully attending to scientific practices involving that concept or notion, and to the language scientists use to express and characterize it, especially in their experimental publications. The basic idea is to set aside all philosophical (metaphysical, epistemological, normative) assumptions about what that concept or notion is supposed to be or do, and try to describe its use by scientists as adequately as possible. The ‘-science’ component of ‘metascience’ is not intended to be honorific, but rather to distinguish this approach from more traditional normative ways of doing the philosophy of science, e.g., ‘metaphysics of science.’ See Bickle (2022) for his most recent attempt to characterize this method. We thank one of our reviewers for anonymously asking us to clarify this term (Bickle, 2022).

^2^This was term of Nagel (1961); numerous other names became popular for these conditions: ‘bridge laws,’ ‘correspondence rules,’…

^3^As one of our reviewers anonymously worried.

^4^Something like this experiment has been done. Using amygdala-dependent tone-fear conditioning and optogenetic manipulation of neuronal activation in mouse lateral amygdala, Rashid et al. (2016) suggested that GABA-releasing (inhibitory) parvalbumin interneurons in lateral and basal amygdala inhibit lateral amygdala neurons from encoding tone-shock memories to control ensemble overlap during memory linking. That study seems to be an actual example of a "circuit-level" control of memory linking mechanisms of the sort we are envisioning here.

^5^These logical and metaphysical conundrums have not been lost on new mechanists. See Craver and Bechtel (2007), and the logical knots they confront trying to make sense of asserting "top down causation without top down causes."

^6^Again, we thank one of our reviewers for anonymously raising this worry for our level-less image.
